# Patterns of Plumericin Concentration in Leaves of *Himatanthus tarapotensis* (Apocynaceae) and Its Interactions with Herbivory in the Peruvian Amazon

**DOI:** 10.3390/plants11081011

**Published:** 2022-04-08

**Authors:** Carlos A. Amasifuen Guerra, Kirti Patel, Piero G. Delprete, Andréa P. Spina, Juan Grados, Pedro Vásquez-Ocmín, Alice Gadea, Rosario Rojas, Jesús Guzmán, Michel Sauvain

**Affiliations:** 1 Laboratorio Mixto Internacional de Química de la Vida, Institut de Recherche Pour le Développement (IRD), Universidad Peruana Cayetano Heredia (UPCH), Avenida Honorio Delgado 430, Urb. Ingeniería, San Martín de Porres 34, Lima 15024, Peru; vasco2224@gmail.com (P.V.-O.); alice.gadea@live.com (A.G.); guzman_j29@hotmail.com (J.G.); michel.sauvain@ird.fr (M.S.); 2 Dirección de Recursos Genéticos y Biotecnología (DRGB), Instituto Nacional de Innovación Agraria (INIA), Avenida La Molina N° 1981, La Molina, Lima 15024, Peru; 3 Unidad de Investigación en Productos Naturales, Laboratorios de Investigación y Desarrollo, Universidad Peruana Cayetano Heredia (UPCH), Avenida Honorio Delgado 439, Urb. Ingeniería, San Martín de Porres 34, Lima 15024, Peru; kirti.patel.fiji@gmail.com (K.P.); rosario.rojas@upch.pe (R.R.); 4 AMAP, IRD, CNRS, CIRAD, INRA, Université de Montpellier, TA A51/PS2, CEDEX 5, 34398 Montpellier, France; piero.delprete@ird.fr; 5 AMAP, IRD, Herbier de Guyane, Cité Rebard, 97300 Cayenne, France; 6 Rua Capitão Leônidas Marques 894, Curitiba 81540-470, Brazil; andreapozetti@gmail.com; 7 Departamento de Entomología, Museo de Historia Natural de la Universidad Nacional Mayor de San Marcos (UNMSM), Av. Gral. Antonio Alvarez de Arenales 1256, Jesús María, Lima 15072, Peru; gradosjuan@hotmail.com; 8 UMR 152 PHARMA-DEV, IRD, Université de Toulouse, CEDEX 9, 31062 Toulouse, France; 9 Laboratorio Centinela de *Helicobacter pylori*, Instituto de Medicina Tropical Alexander von Humboldt, Universidad Peruana Cayetano Heredia (UPCH), Avenida Honorio Delgado 430, Urb. Ingeniería, San Martín de Porres 34, Lima 15024, Peru

**Keywords:** plant-herbivore interactions, plumericin, soil types, precipitation, Peru

## Abstract

We explored the concentration patterns of the bioactive metabolite plumericin produced by *Himatanthus tarapotensis* (Apocynaceae) under different edaphic conditions and variations in rainfall intensity, as well as its potential role in the chemical defense against insect herbivores. Values of plumericin concentration from leaves were obtained by High-Performance Liquid Chromatography, and evaluated as a function of differences in soil types, variation of precipitation, and variation of the abundance of insect herbivores, using first a Repeated Measures Correlation (*rmcorr*) and then a Generalized Linear Mixed Model (GLMM) analysis. Plumericin concentration is highly variable among plants, but with a significantly higher concentration in plants growing on clay soil compared to that of the white-sand soil habitat (*p* < 0.001). Plumericin concentration is not affected by precipitation. The caterpillar of *Isognathus leachii* (Lepidoptera: Sphingidae) is the most conspicuous herbivore of *H. tarapotensis*, and its presence is continuous but not related to plumericin concentration, probably because of its capacity to elude the chemical defense of this plant. Nevertheless, our multivariate model revealed that plumericin concentration is related to the abundance of Hymenoptera (Formicidae), and this relationship is significantly influenced by the soil parameters of carbon percentage, clay percentage, and phosphorous percentage (*p* < 0.001). Plumericin is a mediating agent in the interaction between *H. tarapotensis* and its natural environment. Variation in plumericin concentration would be induced by the abundance of Hymenoptera (Formicidae), probably as a chemical response against these insects, and by differences in soil nutrient availability.

## 1. Introduction

Plant chemistry (i.e., amount and composition of chemical compounds in their tissues) is influenced in part by the variation of environmental factors such as soil nutrient availability [1,2], light intensity [3], carbon dioxide [4], precipitation [5,6], season [7,8], flooding [9], herbivory [10,11], and others. Environmental factors act concomitantly and alter differentially the chemical production of plants [12]. Thus, major hypotheses have been proposed to elucidate variation in phytochemistry under paradigms that consider the simultaneous influence of several environmental factors. The plant-insect coevolutionary theory postulates that variation in plant chemistry is the result of a continuous plant-insect interaction, where the evolution of the chemical defenses in plants is closely followed by the biochemical adaptation of the herbivores [13,14,15]. Considering these plant-insect herbivore interactions, the resource availability hypothesis of plant defense posits that chemical defense strategies of plants (i.e., type and amount of chemical compounds involved in plant defense) vary across soil gradients (i.e., different habitats). A growth-defense trade-off is assumed because plants possess a limited pool of resources that can be invested either in growth or in defense. For example, tissue replacement may cost more for plants in sandy poor nutrient environments (e.g., white-sand soil forest), thus, selecting for higher investment in chemical defenses, in turn, compromises growth rates; by contrast, in higher-resource environments (e.g., clay soil forest) plants would invest more in growth [1,2,16,17,18]. Therefore, soil mosaics can provide an adaptive landscape promoting habitat specialization of plants considering that a trade-off in competitive ability is related to costs of adaptation to soil elements (e.g., calcium, magnesium) [11,19].

The phytochemical landscape hypothesis integrates the mentioned approaches and posits that variation in nutrient availability in soil and variation in trophic interactions (e.g., plant-insect interaction) determine intraspecific variation in plant chemistry across spatial and temporal environmental gradients [20]. The soil mosaic hypothesis [21] mentions that intraspecific variation in plant chemistry is the effect of differences in soil properties (i.e., biotic, chemical, and physical factors), yielding a highly variable phytochemical landscape [20] and leading to cascading ecological and evolutionary effects on higher trophic levels (e.g., herbivores). Plant species and communities are exposed to diverse soil environments across multiple spatial and temporal scales, which can alter the production of metabolites via plasticity and local adaptation, and even lead to divergences in plant populations. Moreover, this variation in phytochemistry can drive herbivore diversification via ecological speciation at fine geographic scales [21]. These relationships could be particularly evident in regions with extreme habitat differences (e.g., white-sand soil forests versus clay soil forests in lowland Amazon).

Thus, resource availability of the soil (e.g., poor nutrient white-sand soil forests versus rich nutrient clay soil forests) determines levels, patterns, and types of chemical production in plants, as well as the nature of the plant-insect herbivores interactions [1,2,20,21]. Moreover, insect herbivores promote the production of specialized toxic substances for plant defense [10,11,22,23,24]. In this case, the degree of dietary specialization in certain plants allows for insect herbivores to find fewer chemical plant defenses and develop adaptations to elude these substances or even to use them [24,25]. Specialist insects feed on only a few related species of plants that share similar chemical traits [13], which are less affected by the chemical defenses of the host plant than are generalists (i.e., insects that consume several plant families). Sequestration has been proposed as an adaptation that predominates in herbivore specialists to elude chemical defenses [26,27]. For example, caterpillars of *Euphydryas phaeton* Drury (Nymphalidae) sequester iridoid glycosides from the herbaceous *Chelone glabra* L. (Plantaginaceae), which is then used to deter predators such as birds [28].

The northwestern portion of the Amazon region is the natural habitat of the tree *Himatanthus tarapotensis* (K. Schum. ex Markgr.) Plumel (Apocynaceae) [29,30]. The latex, bark, and leaves of this plant are used in traditional medicine for the treatment of several diseases such as leishmaniasis, malaria, intestinal parasites, ulcers, infections, and inflammatory processes [31]. These medicinal properties could be related to the presence of the iridoid plumericin, a secondary metabolite isolated from *Himatanthus*, *Plumeria*, and *Allamanda* species of the family Apocynaceae. Plumericin has proven bioactivity in experimental models against leishmaniasis [32], fungi [33,34], bacteria [34,35,36], and tumoral cells [37,38,39]. So far, the role of plumericin in plants remains poorly understood. Only a controlled bioassay has suggested allelopathic properties of this metabolite in the Amazonian species *Duroia hirsuta* (Poepp. & Endl.) K. Schum. (Rubiaceae) [40]. *H. tarapotensis* grows in two types of forests with floristic and edaphic differences, and which are under a permanent attack of insect herbivores feeding on its leaves: white-sand soil forests (WS) poor in nutrients surrounded by more fertile clay, and soil forests (CS) mixed with brown sand. The mosaic distribution of these habitats creates a natural system where the chemistry of plants would be affected by the environmental gradients caused by the soil differences (e.g., differences in soil fertility and insect herbivore community) [2,11,20,21].

The evaluation of interesting bioactive metabolites implies elucidation of their production patterns in nature assuming that their synthesis is influenced by environmental conditions [6,7,41]. In this study, plumericin was extracted from leaves of *H. tarapotensis* and quantified using High-Performance Liquid Chromatography coupled with Diode Array Detector (HPLC-DAD) with the aim of evaluating patterns of plumericin production from *H. tarapotensis* in nature. This evaluation considered edaphic differences between the habitats of this plant, variation in precipitation, and its potential role in the chemical defense against insect herbivores with different degrees of specialization. We investigated the following questions: (1) Is plumericin concentration influenced by differences in soil type? (2) Is plumericin concentration associated with variation in precipitation? (3) Is plumericin concentration positively associated with an abundance of insect herbivores? Considering that edaphic differences originate from a strong environmental gradient of soil fertility affecting the concentration of specialized metabolites in plants (e.g., terpenes) [11,42], we predict that plumericin production of *H. tarapotensis* is affected by differences in soil parameters characterizing these two habitats. In addition, if plumericin is a defense metabolite, its concentration must be positively associated with the abundance of specialist insect herbivores. As the chemistry of plants also shows temporal variation, plumericin production as well as its possible relationship with the abundance of insect herbivores could be influenced by the rainy and dry seasons occurring in the Amazon region [43], because rainfall often influences the composition and abundance of the insect community [6,7,8,44]. Findings will contribute to elucidating the production patterns of bioactive metabolites with high potential in pharmacology, as well as understanding ecological interactions in the Amazon rainforest.

## 2. Results

### 2.1. Variation of Plumericin Concentration and Variation of Environmental Factors: Precipitation, Soil Types, and Insect Herbivores

**Figure 1:**
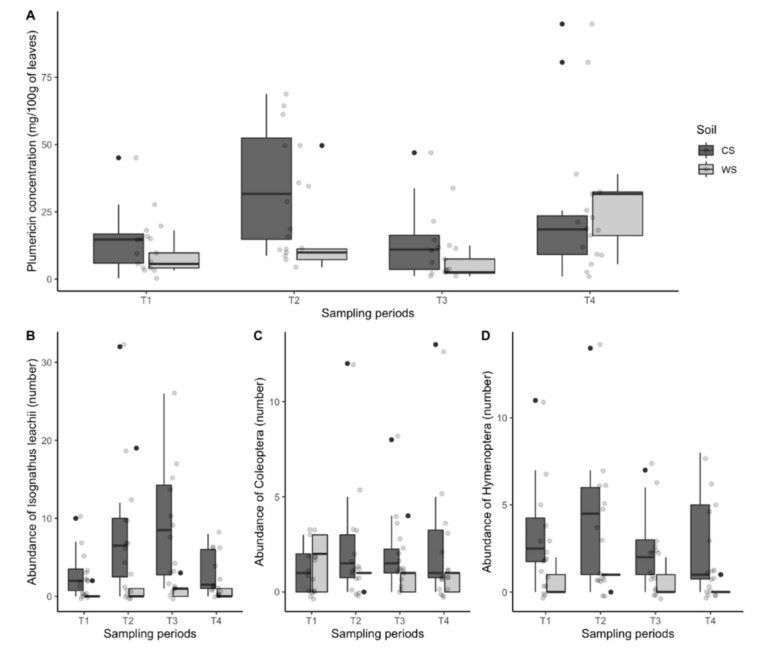
Distribution of the plumericin concentration from 17 plants of *Himatanthus tarapotensis* (**A**) and distribution of the abundance of insect herbivores *Isognathus leachii* (**B**), Coleoptera (**C**), and Hymenoptera (**D**), during the sampling periods (T1: April–June 2014; T2: July–September 2014; T3: October–December 2014; T4: January 2014–March 2015) per soil type (CS = clay soil forest in gray; WS: white-sand soil forest in white). Dots represent the plumericin concentration of 12 plants from CS (in gray) and 5 plants from WS (in white) (**A**) and the abundance of insect herbivores (**B**–**D**) for each plant. In boxplots, the internal horizontal line is the median, the lower border of the box is the 25% percentile, the upper border is the 75% percentile, and external vertical upper and lower lines represent the standard deviation.

Leaves of 17 juvenile plants of *H. tarapotensis* from two Amazonian habitats (white-sand soil forests, WS, and clay soil forests, CS) collected in four samplings between April 2014 and March 2015, have shown a wide range of plumericin concentrations between plants with a median of 12.2 mg/100 g of dry leaves (IQR, 6.1–26.1 mg/100 g of dry leaves) (Figure 1; Table 1) and values ranging from 0.3 to 94.8 mg/100 g of dry leaves (Supplementary Material Table S1). Moreover, plumericin concentration presented temporal significant differences throughout the sampling periods, per soil type and total study area (*p* < 0.001). Two similar peaks of lower concentrations in the first (May 2014) and third (November 2014) samplings were differentiated from two similar peaks of higher concentration during the second (August 2014) and fourth (January 2015) samplings (Figure 1, Table 1).

**Figure 2:**
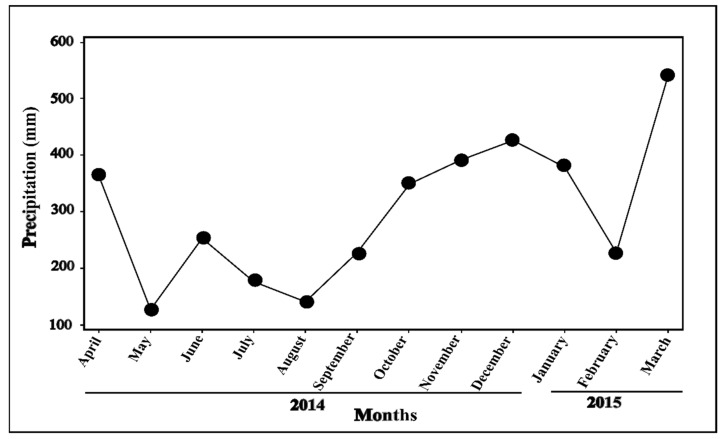
Accumulated monthly precipitation in the study area. The dry season is recorded from May to September 2014 and during February 2015. The rainy season is mainly observed from October 2014 to January 2015. April 2014 and March 2015 are also rainy months. The first (T1: April–June 2014) and fourth (T4; January 2014–March 2015) sampling periods contain dry and rainy months. By contrast, the second (T2: July–September 2014) and third (T3: October–December 2014) sampling periods are included completely during the dry and rainy seasons, respectively (Data Source: meteorological station Moralillos at 12 km from the study area).

Precipitation presented significant variation throughout the sampling periods (*p* < 0.001), with a dry season occurring when the second sampling of leaves was conducted (median: 145.1 mL, IQR: 136.1–286.9 mL), and a greater rainy intensity when the third sampling was realized (median: 418.6 mL, IQR: 370–429.5 mL) (Figure 2, Table 1). These patterns in precipitation are consistent with the temporal rainfall patterns usually occurring in the northern Peruvian Amazon.

Soil types showed significant differences according to edaphic properties such as sand percentage, clay percentage, organic matter percentage, K concentration, and P concentration (*p* < 0.001). More fertile clay soil forests (CS) were distinguished by higher levels of clay percentage, organic matter percentage, and phosphorous percentage in comparison to the poorest white sand soil forests (WS), characterized by higher levels of quarzitic sand (Supplementary Material Tables S2 and S3).

An insect community occurring on *H. tarapotensis* was observed during the study, but the caterpillar of the moth *Isognathus leachii* Swainson (Lepidoptera: Sphingidae) was the most conspicuous herbivore insect feeding permanently on the leaves of this plant. The abundance of this caterpillar was similar among sampling periods for each soil type (CS and WS), however significant variation was observed (*p* < 0.05) when the total study area was considered. A progressive increase in the abundance of *I. leachii* was recorded throughout the first three periods (April–June; July–September; October–December 2014), to a decrease in the fourth period (January–March 2015). Orders Coleoptera (constituted by Anobiidae and Curculionidae) and Hymenoptera (constituted by Formicidae) have also shown a permanent occurrence of *H. tarapotensis* at both WS and CS habitats, but without significant differences throughout sampling periods (Figure 1; Table 1). Several groups of insect visitors were also observed on *H. tarapotensis* during the study but without any relationship with plumericin.

### 2.2. Relationships of Plumericin Concentration with Environmental Factors: Precipitation, Soil Types, and Insect Herbivores

Plumericin concentration was related to environmental factors which would be inducing variation of this metabolite. In the landscape, plumericin wasn’t a factor correlated with precipitation (Table 2), however, this metabolite presented marked differences in concentration between soil types, with a total median of 8.6 mg/100 g of dry leaves for WS, and a nearly double higher total median of 14.9 mg/100 g of dry leaves for CS. This pattern of higher plumericin concentration in CS was observed during the first, second, and third sampling periods, but it was inverse for the fourth sampling when plumericin concentration in WS was nearly double in comparison to CS (Table 1). Plumericin concentration was correlated with specific soil parameters in CS; clay percentage, pH, organic matter percentage, K concentration, and C percentage have shown a positive significant correlation with plumericin, and sand percentage and P concentration presented a negative significant correlation with plumericin (*p* < 0.001). Neither correlation between these soil parameters and plumericin concentration was observed for WS. When the total study area was considered, only pH, P and K concentration, and C percentage were correlated with plumericin concentration (*p* < 0.05), probably caused by the effect of data not correlated from WS. The bivariate regression model considering the two soil types and the four sampling periods as random effects revealed that the specific soil parameters P concentration and C percentage would be inducing an increase in plumericin concentration, with factors of 1.48 and 1.42, respectively. Clay percentage presented a significant *p*-value but with a factor of 0.87, which means that the increase in clay percentage would be associated with a decrease in plumericin concentration (Table 3A). Due to their significant relationship with plumericin concentration, these three soil parameters were later considered as fixed effects for the bivariate and multivariate models considering insect herbivores as covariates.

Plumericin concentration was also positively correlated with the abundance of the insect herbivore *I. leachii*, but only for WS, and with Coleoptera (constituted by Anobiidae and Curculionidae) for CS and the total study area (*p* < 0.05) (Table 2). However, when the regression bivariate and multivariate models were performed, neither relationship was observed between plumericin concentration and the abundance of *I. leachii*. Moreover, although Coleoptera presented significant relationships with plumerin concentration, values of magnitude expressed as factors less than one indicate that the increase in abundance of Coleoptera would be associated with a decrease in plumericin concentration. Finally, Hymenoptera (Formicidae) presented a regression value greater than one, which was significant when the multivariate regression was performed with a C percentage as a fixed effect (Table 3B). It could be preliminarily claimed that variation in plumericin concentration in the study area is induced by the abundance of the insect herbivores Hymenoptera (Formicidae), and this relationship is significantly influenced by soil parameters mainly by C percentage.

## 3. Discussion

**Figure 3:**
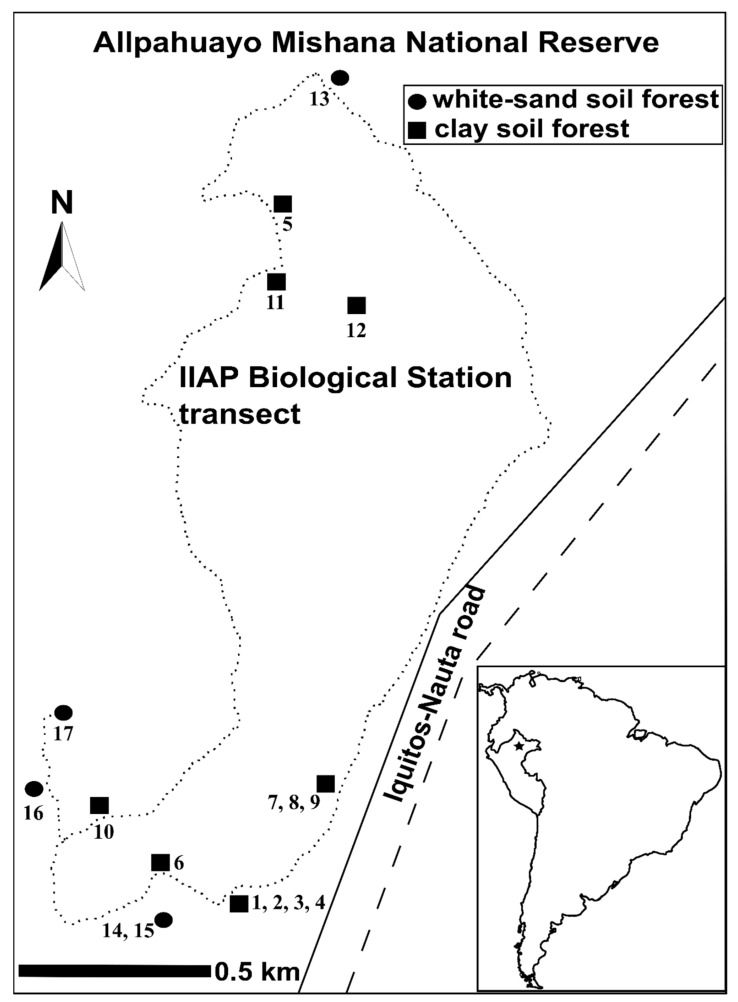
Map showing the location of the study area and the position of the sampling sites. Dotted line is the sampling transect. Circles refer to plants situated on the white-sand soil forest, and squares are plants situated in clay soil forest. Numbers are the codes of the evaluated plants.

Evaluation of chemical variation is critical to understanding the production patterns of bioactive metabolites of plants. In Amazon forests, plumericin concentration from *H. tarapotensis* would be influenced by differences in soil nutrients between the habitats CS and WS, confirming other study findings revealing that differences in availability of soil resources significantly influence the chemical investment of plants [1,2,45]. Differences in plumericin concentration between the two soil types occurred even for neighboring plants and grouped spatially to the north or the south of the study area (Figure 3). This means that differences in soil characteristics would be a critical factor affecting plumericin production in *H. tarapotensis* even at a stand scale. Plant chemistry is in part a function of soil chemistry (i.e., soil nutrients) on the phytochemical landscape [20]. Soil attributes (i.e., chemical and physical factors) can alter secondary metabolites via plasticity and local adaptation of plants across the soil mosaic on the landscape [21]. For example, in the phytochemical landscape, differences among habitats in soil nutrient availability can shape the genetic architecture of plant populations and the molecular machinery by which plants take up nutrients from soils [20]. Our findings suggest that differences in soil attributes have effects on plumericin concentration in *H. tarapotensis*, and clay percentage, C percentage, and K concentration, would be the main soil parameters promoting differences of plumericin concentration between habitats, and potentially of the chemistry of this plant.

In addition, higher levels of plant chemical defense are expected in environments with limited soil resources (e.g., WS) [2], and mainly carbon-based secondary metabolites (e.g., terpenes) [1]. So, if plumericin is a defense metabolite, a higher concentration of it would be expected in WS. However, in the study area, plumericin concentration was particularly higher in CS, and only during the fourth sampling median value in WS was nearly double in comparison to CS. It has been claimed that the level of defense in a poor environment is not only determined by the environment but is also the result of the plant’s adaptation to this environment [20,21,46]. Indeed, a plant species that is not completely adapted to poor environments may be more attractive to herbivores [46]. It could be the case of plumericin in *H. tarapotensis*, considering that insect herbivores have shown a permanent occurrence at both CS and WS, but more in-depth studies are necessary to evaluate this hypothesis.

Plumericin concentration also showed temporal variation throughout the study, with higher interspersed with lower median values at both CS and WS (Figure 1A, Table 1). Temporal variation of metabolites and/or compounds has been frequently associated with the periodicity of rainfall. In the Neotropics, the relationship between specialized metabolites concentration and precipitation has mostly been focused on ecosystems with higher seasonality (e.g., central Amazon, Brazilian Cerrado, Atlantic forest). In various studies, concentration rates of compounds (e.g., tannins, sesquiterpenes, essential oils) or specific metabolites (e.g., emodin, goyazensolide) have been positively or negatively correlated with precipitation seasonality [6,7,8,44]. However, other studies did not find any correlation between certain compounds (e.g., tannins, sesquiterpenes, lactones) and precipitation intensity (i.e., dry or rainy season; [47,48]). In our study area, precipitation intensity showed significant variation (*p* < 0.001) which normally occurs in the northern Peruvian Amazon [43]. Yet, rainfall variations were not correlated with the temporal variation of plumericin concentration (Table 2).

Moreover, a particular insect community visiting *H. tarapotensis* at CS and WS was observed. The caterpillar of *Isognathus leachii* (Lepidoptera: Sphingidae) was the most conspicuous herbivore permanently feeding leaves of *H. tarapotensis* in both habitats. Variations in the abundance of *I. leachii* were significant and positively correlated with the plumericin concentration only for WS (*p* < 0.001) (Table 2), but neither relationship was observed when the bivariate and multivariate GLMM models were performed. Plumericin concentration would be not affected by the presence of the herbivore *I. leachii* and its continuous presence on *H. tarapotensis* could be explained by the mechanism of high specificity (i.e., probably mutualistic) between sphingid caterpillars and plants species mainly of the family Apocynaceae [49,50,51]. Species of sphingid caterpillars have the capacity to sequester and accumulate toxic compounds (e.g., iridoids), derived from the host plant to protect themselves against natural predators [52,53,54]. Although the presence of these toxic compounds in the insect herbivores was not analyzed in this study, if plumericin is part of the chemical defense of *H. tarapotensis*, we hypothesized that the caterpillar of *I. leachii* is able to sequester and accumulate its defense compounds, including the iridoid plumericin.

Insects from the orders Coleoptera (Anobiidae and Curculioniidae) and Hymenoptera (Formicidae) were also recorded continuously on *H. tarapotensis* in WS and CS habitats. Both coleopteran families Anobiidae and Curculionidae are phytophagous associated with Neotropical plant species [11], which includes Apocynaceae. The positive and significant correlation between plumericin concentration and Coleoptera highlights the potential role of this metabolite in chemical defense. However, experimental studies are necessary to evaluate this relationship, considering that when the bivariate and multivariate regression models were performed, Coleoptera presented significant relationships with plumericin concentration but with values of magnitude expressed as a factor less than one. Thus, the increase in the abundance of Coleoptera would be associated with a decrease in plumericin concentration. For Hymenoptera (Formicidae), correlation with plumericin concentration was not observed, but when the multivariate GLMM was performed a significant relationship was recorded with a magnitude factor of 1.09 (CI: 1.01–1.20). So, it could be claimed that the abundance of Hymenoptera induces variation in plumericin concentration, but in this case, experimental studies are also necessary. Other insect groups showed an intermittent occurrence of *H. tarapotensis* as eventual insect visitors without any relationship with the plant.

Our preliminary results contradict any participation of plumericin as an anti-herbivore agent against the specialist herbivore caterpillar *I. leachii*. However, the significant relationship between plumericin concentration and the abundance of coleopterans and hymenopterans reveals a potential function of this metabolite as a deterrent against other insect herbivores. It has been reported that the variation of concentration of a single terpene (e.g., cariophyllene, α-muurolene, selinene, cadinene, ocimene, aucubin) from the compound mixture for the chemical defense of plants can have a significant effect against herbivory, and in turn herbivory could cause the variation in concentration of a single compound [10,22]. Although only *I. leachii* was observed causing evident mechanical damage (e.g., eaten areas) in leaves of *H. tarapotensis*, it is probable that coleopterans and hymenopterans would attack leaf meristems and other parts of the leaves and suck the phloem from *H. tarapotensis*. This would be causing damage but without visible marks on the leaf blades [11]. Although this metabolite has no effect against *I. leachii*, it could participate in the chemical defense of *H. tarapotensis* against other insect herbivores. We conclude that there is no relationship between plumericin concentration and the abundance of the herbivore *I. leachii*, probably because of the capacity of this caterpillar to elude the chemical defense of *H. tarapotensis*. Future studies could test whether plants with high concentrations of plumericin repel other insects besides the sphingids. We predict that the individuals of *H. tarapotensis* with a low concentration of plumericin would suffer a greater attack from other herbivore species.

In the environment, nutrient availability is a critical factor that provides a mechanism linking nutrient dynamics with trophic interactions through the nexus of the phytochemical landscape. At the local scale, nutrient availability and herbivory both influence the range of the chemical profile that plants express [20,21]. In the study area, soil properties and abundance of insect herbivores would be the main factors influencing the variation in plumericin concentration of *H. tarapotensis* in the phytochemical diverse landscape.

The study of specialized metabolites and their ecological consequences are relevant not only for understanding how plants interact with their environment but also as an ecological base for the study of bioactive compounds for the benefit of human health. Plumericin is a bioactive iridoid with high potential in pharmacology, and our results contribute to elucidating the ecological aspects related to its concentration in *H. tarapotensis*. Variables and patterns affecting plumericin concentration were evident and should not be neglected. It is possible that a larger sampling may have provided more evidence of the phenomena, however, the field and laboratory work were exhaustive in order to obtain the most accurate and representative data possible. Moreover, it is necessary to consider that the study was developed under the natural conditions of the Amazonian rainforest where simultaneously various abiotic and biotic factors could be involved and affect the chemical production of *H. tarapotensis*. Finally, understanding the interactive effects of secondary metabolites on trophic interactions should be a high priority for future research. So, we hope that our findings will stimulate the development of further studies analyzing the multiple ecological functions that influence specialized metabolites production and concentration in plants, to be performed by multidisciplinary teams integrated by ecologists, chemists, biochemists, molecular biologists, entomologists, and taxonomists.

## 4. Materials and Methods

### 4.1. Study Area

This study was carried out in the northwestern part of the Amazonian lowlands, within the Biological Station of the Instituto de Investigaciones de la Amazonía Peruana (IIAP), located in the Allpahuayo Mishana National Reserve (RNAM) about 20 km south of the city of Iquitos in northern Peru, between the coordinates 3°57–58′ S, 73°25′ W and 107–165 m of altitude (Figure 3). In this locality, 737 juvenile plants from a population of *H. tarapotensis* are distributed along a circuit of ca. 9 km, where 172 were found on white-sand soil forests and 565 in clay soil forests.

### 4.2. Precipitation

In the study area, the precipitation reaches daily values higher than 3000 mm during the year, with monthly values that do not fall below 100 mm. Normally, a dry season occurs between June and August, and the rainy season begins at the end of August until the beginning of June, with a maximum between February and April [43]. Data of the monthly accumulated values of precipitation were registered throughout the study from April 2014 to March 2015, in order to explore variation in rainfall (Figure 2). This data was obtained from the nearby meteorological station Moralillos (3°53′59′′ S, 73°20′17′′ W) situated 12 km from the study area.

### 4.3. Soil Types

The study area is characterized by having two main habitats with floristic and edaphic differences, the white-sand soil forests (WS) which are surrounded by contiguous clay soil forests (CS) [11,42]. Seven representative soil samples collected simultaneously at the beginning of the study and previous to the first sampling of plant material, were used to evaluate differences in physical-chemical soil parameters between WS and CS. Soil collection was spatially distributed according to the distribution of plants of *H. tarapotesis*. Two samples of superficial soil (0–15 cm depth) from WS and five samples collected on CS were used to measure: soil texture (percentages of sand, silt, and clay), pH, electric conductivity, percentage of organic material, phosphorus (P), potassium (K), the capacity of cationic interchange, interchangeable cations (Ca^2+^, Mg^2+^, K^+^, Na^+^, Al^3+^, H^+^), cation sum, the sum of cation bases, percentage of base saturation, and carbon percentage (C) (Supplementary Material Table S2).

### 4.4. Studied Plants

Plumericin was evaluated from the leaves of 17 juvenile plants of the *H. tarapotensis* population occurring along the forest circuit and grouped to the south and north of the study area. For selecting the plants, firstly we identified all juvenile plants less than 5 m in height in order to guarantee the evaluation of plants of similar age. Then, only plants with foliage available for a periodical and continuous (every three months) sampling of leaves were recorded. All juvenile plants accomplishing the above criteria and occurring at WS and CS were coded using a successive numbering (from 1 to 17). Thirteen plants were recorded to the south (WS: 4; CS: 9) and four to the north (WS: 1; CS: 3) of the study area (total = WS: 5; CS: 12) (Figure 3). In order to monitor the normal growth of plants, the height was measured periodically at the beginning of the study (April 2014), followed by another three measurements, in July and November 2014, and March 2015. Plants presented an initial height ranging between 97 and 417 cm, with a maximal difference in height of 320 and 428 cm at the beginning and at the end of the study, respectively. The size of the plants is supplied in Supplementary Material Table S4.

To achieve a taxonomical identification of the plants, fertile samples from the mother trees were identified, and their vouchers: C.A. Amasifuen, M. Shupingahua and O. Pizuri 3473 (USM); C.A. Amasifuen and R. Pua 3481 (USM); C.A. Amasifuen and R. Pua 3483 (AMAZ, MBM); and C.A. Amasifuen and R. Pua 3487 (MBM) were deposited at the AMAZ (Iquitos) and USM (Lima) herbaria in Peru, and at the MBM (Curitiba) herbarium in Brazil.

### 4.5. Herbivory and Insect Community

Herbivory was evaluated by measuring the abundance of the insect herbivores feeding on the 17 juvenile plants of *H. tarapotensis*, from April 2014 to March 2015. This procedure was based on the original method developed by Fine et al. [11]. Insect communities occurring on the studied plants were recorded, and those feeding on the plants were differentiated from those merely visiting. This procedure was used to prevent a potential evaluation bias related to the quantification of mechanical damage on leaves because entirely eaten leaves and damaged leaves without apparent foliar area loss are difficult to quantify [11,23]. Insect evaluation was carried out under similar conditions for all the evaluated plants. Insects were collected manually or using an entomological aspirator. Sampling was realized at 15 days intervals, between 8.00 and 14.00 h, with an average collection time of 15 min per plant. A meticulous observation was realized about the feeding behavior of the recorded insects, in order to recognize potential specialists and/or generalists. Then, insects were identified at the taxonomic level of the family and later differentiated as morpho-species. The corresponding voucher specimens are deposited at the Museo de Historia Natural de la Universidad Nacional Mayor de San Marcos (UNMSM), Entomology Department, Lima, Peru, as C.A. Amasifuen, N. Macedo, J. Inuma and P. Guedes sp498. The monthly quantity of insects was calculated for the 17 plants evaluated. The abundance (i.e., number of individuals) of the specialist insect herbivore recorded on *H. tarapotensis* is summarized in Supplementary Material Table S5.

### 4.6. Sampling of Plant Material

Approximately 20 g of fresh and intact mature leaves (12–15 spatially representative leaves of the foliage) from each of the 17 juvenile plants, were collected every three months, almost simultaneously with the measurement of the height of the plants as described above. Leaves were collected throughout the short dry season (12 May and 8 August 2014) and the rainy seasons (12 November 2014 and 31 January 2015) occurring in the north of the Peruvian Amazon [43]. The number of leaves to collect and the periodicity of the collection were established to minimize the stress on plants as well as to guarantee the minimum necessary quantity of leaves to perform the chemical tests. Samplings of leaves were realized during the same daytime intervals of sampling of insects (i.e., between 8.00 and 14.00 h), on sunny days (without rain), with temperatures of 26–28.1 °C. All plants were found growing under similar luminosity conditions on small gaps or towards the edge of the forest, considering that *Himatanthus* species are heliophilics [29]. Similar conditions for sampling contributed to reducing any bias of the compound quantification [7,23,44,47]. Only intact leaves were collected, considering that those attacked by the herbivores were completely consumed (field observation). The collection of intact leaves is useful to study the relationship between the production of chemical compounds and herbivory, considering that defense compounds are induced at the level of the affected organs (i.e., the leaves), and not only in part of it [11,23].

The collected leaves were immediately dried in driers with forced air circulation at 40 °C for 7 days until their dry weight was stabilized. The dried leaves were then stored in hermetic bags, preserved at 20 °C, away from solar exposure, and free from insects.

### 4.7. Samples Preparation

After storage, the leaves were again dried at 40 °C in a drier for 24 h, and later pulverized. The extraction method has been modified from the original method of Silva et al. [55]. For each pulverized leaf sample, 250 mg was diluted in 5 mL of methanol:water (1:1 *v*/*v*) solution followed by sonication for 1 h with vortex agitations at 15 min intervals, to obtain a homogeneous dilution, which was then centrifuged at 5000 rpm for 10 min. The supernatant was transferred into a 10 mL vial and the pellet was submitted to the same method of sonication, vortex, and centrifugation. The second supernatant was transferred to the same 10 mL vial and made to the volume with methanol/water solution. This supernatant solution was passed through a 45 µm Millipore filter before HPLC-DAD analysis. From each pulverized leaf sample, two replicates of 250 mg each were submitted for extraction.

### 4.8. Analytical Conditions for HPLC Method

Plumericin detection in extracts and in standard samples was performed using an HPLC-DAD device (ELITE LaChrom). A gradient system developed in the lab was applied: solvent A (0.05% Trifluoroacetic acid (TFA) in water milli-Q, and solvent B (acetonitrile) for 30 min on a C18 column (Merck©, France; column temperature 27 °C) at a flow rate of 1.0 mL/min. The Elution profile: 0 to 7 min, 40 to 45% B (linear gradient); 7 to 12 min, 45% B (isocratic); 12 to 13 min, 45 to 50% B (linear gradient); 13 to 15 min, 50 to 70% B (linear gradient); 15 to 17 min, 70 to 100% B (linear gradient); 17 to 28 min (column washing), 100% B (linear gradient). Plumericin detection and quantification were established at 230 nm wavelength.

### 4.9. Analytical Curve (Linearity) of Standard

**Figure 4:**
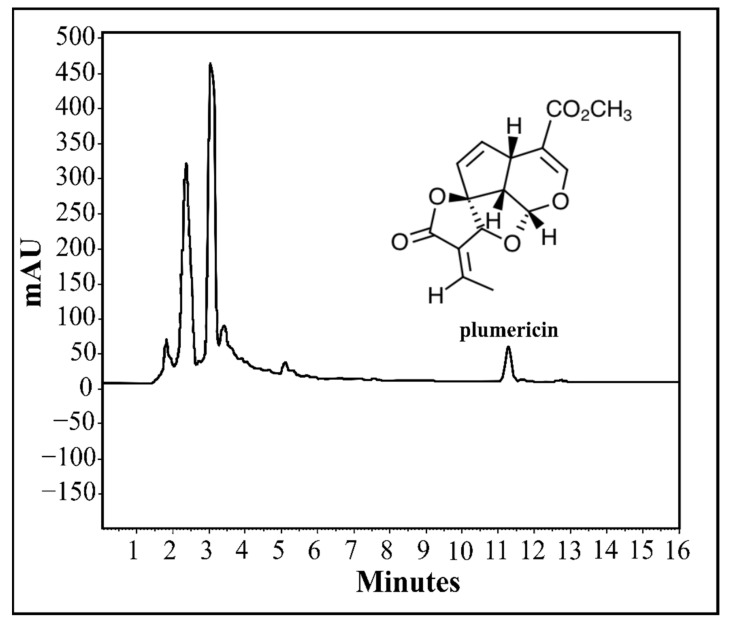
HPLC-DAD chromatogram (wavelength λ = 230 nm) for the crude extract of *H. tarapotensis* leaves and structure of plumericin.

Plumericin was isolated from *H. tarapotensis*, identified with NMR (^1^H and ^13^C), and purity was checked by HPLC-DAD. HPLC chromatogram and structure of plumericin are shown in Figure 4. A stock solution was prepared by dissolving 2.2 mg of pure plumericin in 5 mL of acetonitrile to obtain a concentration of 0.44 mg/mL. Then, five working standard solutions of plumericin were prepared by appropriate dilution of the stock solution with acetonitrile to generate concentrations ranging from 4.40 × 10^−3^ mg/mL to 0.28 × 10^−3^ mg/mL for the standard calibration curve. For each dilution, 5 µL was injected into the HPLC. The analyses were realized in triplicates, and the peak areas (AUC, areas under the curve) obtained were used to elaborate the calibration curve from linear regression with a correlation coefficient of R^2^ > 0.998.

### 4.10. Detection and Quantification of Plumericin

Detection of the chromatographic peak from plumericin in *H. tarapotensis* extracts was established by co-injection of the plumericin standard, verifying the retention time and UV spectrum of the compound. To quantify plumericin from *H. tarapotensis* extracts, each sample was injected at 5 µL in duplicate. Plumericin concentration of each extract was estimated as an average ratio of the two peak areas, analyzed in relation to the calibration curve obtained with the standard compound. Quantities were expressed in milligrams (mg) of plumericin per 100 g of dry leaves from *H. tarapotensis*.

### 4.11. Validation of the Analytical Method

The analytical method was validated according to the guidelines of the International Council for Harmonization [56]. The calibration curve presented an acceptable correlation coefficient (R^2^ = 0.9981). Minimal values of detection limit (LOD = 0.00027 mg/mL) and quantification (LOQ = 0.0082 mg/mL) were appropriate to detect and quantify plumericin. Variation coefficients (% CV) were satisfactory based on the evaluation of two random extracts (sample 1 = 0.6; sample 2 = 3.1). In addition, the robustness analysis demonstrated that this method is reliable and stable, assuming that the measurements of the concentration of plumericin were not affected by deliberate changes in the flux and temperature of the column.

### 4.12. Statistical Analyses

We analyzed the relationship of the plumericin concentration from *H. tarapotensis* with environmental factors such as soil, precipitation, and insect herbivores (*Isognathus leachii*, Hymenoptera constituted by Formicidae, and Coleoptera constituted by Anobiidae and Curculionidae) using Generalized Linear Mixed Models (GLMMs) [57].

Previously, differences in plumericin concentration and insect abundance were tested between the two soil types of the study area (WS and CS), and among four sampling periods (T1: April–June 2014; T2: July–September 2014; T3: October–December 2014; T4: January 2014–March 2015). We performed central tendency statistics, and the variances were calculated with the non-parametric Friedman test because the data did not have a normal distribution. In order to evaluate significant differences, values of each variable were compared in pairs using the *post hoc* Wilcoxon test, and a Bonferroni test was used for fitting the significance level due to the effects of multiple comparisons.

On the other hand, in order to identify edaphic properties characterizing soil types, quantitative differences in soil parameters between WS and CS were evaluated using a Student *t*-test. Five parameters (sand percentage, clay percentage, organic matter percentage, K concentration, and P concentration) showed significant differences between WS and CS (Supplementary Material Table S3), and two other parameters (pH and C percentage) that are usually used to characterize and distinguish Amazonian soils were selected to explore the relationship of the soil with plumericin concentration.

Then, we evaluated the relationship of plumericin concentration with the abundance of insect herbivores, precipitation, and the seven soil parameters selected. First, a Repeated Measures Correlation (*rmcorr*) was performed in order to explore relationships of plumericin concentration with each variable, per soil type and total study area. This procedure allowed us to identify insect herbivores and soil parameters significantly related to the plumericin concentration.

In order to quantify the magnitude of the relationships observed with *rmcorr*, a bivariate GLMM was performed between plumericin concentration and the insect herbivores and soil parameters, considering the two soil types and the four sampling periods as random effects. Finally, a multivariate GLMM was performed in order to evaluate simultaneously the effects of covariates insect herbivores, soil parameters, and precipitation on plumericin concentration. In the multivariate regression model, the response variable was plumericin concentration, precipitation, and soil parameters (clay percentage, C percentage, and P concentration) significantly related to plumericin concentration according to the bivariate model, were considered as fixed effects, the plant was the random effect, error distribution was expressed by a gamma distribution, and link-function was a log. This procedure was established considering that in nature several variables could affect simultaneously plumericin concentration.

Analyses were conducted on the statistical programming language R version 1.3.1073 using the *rmcorr* and *lme4* packages. Figures were created with Inkscape 0.92 (Software Freedom Conservancy Inc., New York, NY, USA).

## Figures and Tables

**Table 1:** Median values and interquartile ranges (IQR) of the variables: plumericin concentration, precipitation, and abundance of insect herbivores, per soil type and sampling period (CS = clay soil forest; WS = white-sand soil forest; T1: April–June 2014; T2: July–September 2014; T3: October–December 2014; T4: January 2014–March 2015).

Variable			Total	Sampling Periods				
				T1	T2	T3	T4	*p ^a^*
			Median (IQR)	Median (IQR)	Median (IQR)	Median (IQR)	Median (IQR)	
**Plumericin** **mg/100 g of leaves**			12.2 (6.1–26.1)	9.8 (4.4–15.8)	18.6 (10.9–45.5)	7.5 (3.4–12.5)	18.8 (9.3–31.7)	<0.001
	**CS**		14.9 (8.9–27.9)	14.7 (5.9–16.8)	31.7 (14.8–52.5)	10.9 (3.6–16.3)	18.5 (9.2–23.5)	<0.001
	**WS**		8.6 (4.4–16.7)	5.7 (4.2–9.8)	9.9 (7.3–11.2)	2.6 (2.2–7.5)	31.7 (16.2–32.4)	<0.001
**Precipitation** **(mL)**			326.9 (145.1–421.3)	190.2 (119.8–390.1)	145.1 (136.1–286.9)	418.6 (370–429.5)	464.3 (186.5–546.2)	<0.001
**Insect herbivores** **(number)**								
	**Lepidoptera** (***Isognathus leachii***)		2 (1–7)	2 (0–3)	4 (1–10)	4 (1–10)	1 (1- 6)	0.011
		**CS**	0.5 (0–1)	2 (1–3.5)	6.5 (2.8–10)	8.5 (2.8–14.3)	1.5 (1–6.3)	
		**WS**	3.5 (1–8)	0 (0–0)	0 (0–1)	1 (0–1)	1 (1–1)	
	**Hymenoptera**		1 (0–3.3)	2 (0–3)	1 (1–5)	1 (0–2)	1 (0–3)	0.136
		**CS**	2 (1–5)	2.5 (1.8–4.3)	4.5 (1 -6)	2 (1–3)	1 (0.8–5)	
		**WS**	0 (0–1)	0 (0–1)	1 (1–1)	0 (0–1)	0 (0–0)	
		**Coleoptera**	1 (0–2)	1 (0–2)	1 (1–2)	1 (1–2)	1 (0–2)	0.791
		**CS**	1 (0–1)	1 (0–2)	1.5 (0.8–3)	1.5 (1–2.3)	1 (0.8–3.3)	
		**WS**	1 (0.8–2.3)	2 (0–3)	1 (1–1)	1 (0–1)	1 (0–1)	

^a^ Friedman test *p*-value. *p* values measure the significance of temporal variations among sampling periods.

**Table 2:** Repetitive Measurement Correlation Analysis (*rmcorr*) between plumericin concentration and environmental variables: insect herbivores, precipitation, and soil parameters (CS = clay soil forest; WS = white-sand soil forest).

			Total			CS			WS		
			r_rm_	95%CI	*p* ^a^	r_rm_	95%CI	*p* ^a^	r_rm_	95%CI	*p* ^a^
**Insect herbivores** **(number)**											
	**Lepidoptera**		0.027	−0.253–0.303	0.848	−0.165	−0.472–0.177	0.328	0.746	0.356–0.914	<0.001
		***Isognathus leachii***									
	**Hymenoptera**		−0.109	−0.376–0.174	0.440	−0.085	−0.407–0.255	0.615	−0.254	−0.691–0.318	0.341
		**Formicidae**									
	**Coleoptera**		0.295	0.018–0.529	0.033	0.357	0.027–0.617	0.029	−0.047	−0.563–0.495	0.862
		**Anobiidae**									
		**Curculionidae**									
**Precipitation** **(mL)**			−0.136	−0.399–0.147	0.333						
**Soil parameters**											
	**Sand percentage**		0.146	−0.137–0.408	0.301	0.485	0.182–0.704	0.002	0.080	−0.470–0.585	0.767
	**Clay percentage**		−0.220	−0.470–0.061	0.115	−0.492	−0.709–−0.191	0.001	0.019	−0.516–0.544	0.943
	**pH**		−0.403	−0.612–−0.140	0.003	−0.574	−0.761–−0.297	0.001	0.112	−0.444–0.607	0.677
	**Organic matter percentage**		−0.005	−0.283–0.272	0.966	−0.567	−0.757–−0.289	0.001	0.052	−0.492–0.567	0.847
	**Phosphorous ppm**		0.405	0.143–0.614	0.002	0.574	0.298–0.7619	0.001	−0.107	−0.603–0.449	0.693
	**Potassium ppm**		−0.314	−0.544–−0.039	0.023	−0.551	−0.747–−0.267	0.001	0.347	−0.224–0.741	0.186
	**Carbon percentage**		0.435	0.178–0.636	0.001	0.559	0.277–0.752	0.001	0.088	−0.463–0.591	0.743

^a^*rmcorr* test *p*-value.

**Table 3:** Estimated average of GLMM of the relationship between plumericin concentration of *Himatanthus tarapotensis* associated with environmental factors: (**A**) soil parameters, (**B**) insect herbivores.

**(A)**					
**Soil Parameters**			**Bivariate Analysis**		
			**Mean**	**95%CI**	
	**Sand percentage**		1.02	0.96–1.07	
	**Clay percentage**		0.87 *	0.71–0.98	
	**pH**		0.69	0.23–1.09	
	**Organic matter percentage**		0.85	0.63–1.15	
	**Ppm Phosphorous**		1.48 *	1.09–1.72	
	**Ppm Potassium**		0.83	0.54–1.39	
	**Carbon percentage**		1.42 **	1.29–1.61	
**(B)**					
		**Bivariate Analysis**		**Multivariate Analysis**	
**Insect Herbivores**		**Mean**	**95%CI**	**Mean**	**95%CI**
**Lepidoptera**					
	***Isognathus leachii***	1.01	0.97–1.05	1.01 ^a^	0.99–1.03
				1.01 ^b^	0.99–1.02
**Hymenoptera**					
	**Formicidae**	1.09	0.99–1.21	1.07 ^a^	0.96–1.19
				1.09 ^b^*	1.01–1.20
**Coleoptera**					
	**Anobiidae**	0.17 ***	0.04–0.62	0.17 ^a,^***	0.05–0.57
				0.18 ^b,^***	0.05–0.56
	**Curculionidae**	0.52 ***	0.33–0.80	0.53 ^a,^***	0.33–0.80
				0.51 ^b,^**	0.34–0.76

* *p* < 0.05; ** *p* < 0.01; *** *p* < 0.001. ^a^ Multivariate model 1: fitted by clay percentage, carbon percentage, phosphorus concentration and precipitation. ^b^ Multivariate model 2: fitted by carbon percentage.

## Data Availability

All data generated is provided in this manuscript.

## Supplementary Materials

The following are available online at https://www.mdpi.com/article/10.3390/plants11081011/s1, Table S1: Plumericin concentration from leaves of juvenile plants of *Himatanthus tarapotensis* collected in clay soil forests (CS) and white-sand soil forests (WS) during the dry and rainy season of the Amazon, Table S2: Values of 18 physical-chemical variables of superficial soil (0–15 cm depth) of clay soils (CS) and white-sand soils (WS) from the IIAP Biological Station (CE = electric conductivity; P = phosphorus; K = potassium; CEC = cation-exchange capacity; Ca = calcium; Mg = magnesium; Na = sodium; Al = aluminum; H = hydrogen; C = carbon), Table S3: Student *t*-test for comparison of physical-chemical parameters of superficial soil (0–15 cm depth) from clay soil forests (CS) and white-sand soil forests (WS) of the study area, Table S4: Height (cm) of individual plants of *Himatanthus tarapotensis*, per month of evaluation (Apr = April; May = May; Jun = June; Jul = July; Aug = August; Sep = September; Oct = October; Nov = November; Dec = December; Jan = January; Feb = February; Mar = March), Table S5: Abundance (number of individuals) of the insect herbivore *Isognathus leachii*, per individual plants of *Himatanthus tarapotensis* and month of evaluation (Apr = April; May = May; Jun = June; Jul = July; Aug = August; Sep = September; Oct = October; Nov = November; Dec = December; Jan = January; Feb = February; Mar = March).
