# Supplementary material for: Patterns of Plumericin Concentration in Leaves of Himatanthus tarapotensis (Apocynaceae) and Its Interactions with Herbivory in the Peruvian Amazon
Source: Plants (Basel). 2022 Apr 8;11(8):1011. doi: 10.3390/plants11081011 (PMC9027084; doi:10.3390/plants11081011)
Supplement: Supplementary file 1 [file plants-11-01011-s001.zip › plants-1637133-supplementary.pdf]

SUPPLEMENTARY MATERIAL

TABLE S1 Plumericin concentration from leaves of juvenile plants of *Himatanthus tarapotensis* collected in clay soil forests (CS) and white-sand soil forests (WS) during the dry and rainy season of the Amazon

Specimen Number	Habitat	Plumericin concentration (mg/100 g of dry leaves) (mean values ± standard deviation)			
		Precipitation periods			
		Dry		Rainy	
		**May 2014**	**August 2014**	**November 2014**	**January 2015**
01	Clay soil forest (CS)	0.30 (± 0.01)	10.90 (± 0.97)	1.14 (±0.06)	0.94 (± 0.03)
02		9.49 (± 0.55)	8.73 (± 0.34)	21.51 (± 1.88)	11.93 (± 0.25)
03		4.36 (± 0.18)	18.59 (± 0.45)	3.71 (± 0.13)	2.63 (± 0.19)
04		27.73 (± 2.05)	64.39 (± 1.22)	14.61 (± 0.12)	22.82 (± 0.85)
05		19.74 (± 1.26)	49.54 (± 2.80)	46.96 (± 2.77)	94.79 (± 0.82)
06		14.86 (± 0.56)	61.21(± 3.14)	6.25 (± 0.10)	21.27 (± 1.10)
07		15.81 (± 1.13)	34.58 (± 1.11)	3.36 (± 0.06)	18.81 (± 0.86)
08		15.07 (± 1.71)	15.87 (± 1.66)	11.36 (± 0.34)	9.25 (± 1.06)
09		14.58 (± 0.47)	35.77 (± 0.16)	3.41 (± 0.23)	18.15 (± 1.32)
10		6.48 (± 0.20)	11.53 (± 0.13)	10.60 (± 0.71)	25.60 (± 1.11)
11		3.31 (± 0.38)	28.80 (± 2.51)	11.87 (± 0.51)	8.94 (± 0.49)
12		45.08 (± 2.05)	68.76 (± 1.15)	33.80 (± 0.89)	80.56 (± 2.51)
13	White-sand soil forest (WS)	5.67 (± 0.52)	49.63 (± 2.34)	1.14 (± 0.03)	5.54 (± 0.29)
14		3.22 (± 0.07)	9.93 (± 0.41)	2.56 (± 0.17)	31.66 (± 1.97)
15		4.15 (± 0.40)	7.25 (± 0.18)	12.54 (± 1.37)	16.21 (± 0.46)
16		18.12 (± 0.79)	4.46 (± 0.18)	2.19 (± 0.07)	39.02 (± 0.33)
17		9.75 (± 0.35)	11.21 (± 0.42)	7.48 (± 0.69)	32.39 (± 0.46)

TABLE S2 Values of 18 physical-chemical variables of superficial soil (0–15 cm depth) of clay soils (CS) and white-sand soils (WS) from the IIAP Biological Station (CE = electric conductivity; P = phosphorus; K = potassium; CEC = cation-exchange capacity; Ca = calcium; Mg = magnesium; Na = sodium; Al = aluminum; H = hydrogen; C = carbon)

**Physico-chemical variables**		**Sampling sites**						
		**White-Sand Soil**		**Clay Soil**				
		**01**	**02**	**03**	**04**	**05**	**06**	**07**
Sand / %		87	89	41	63	49	51	53
Silt / %		5	5	41	27	35	37	31
Clay / %		8	6	18	10	16	12	16
pH (1:1)		4.60	4.19	4.10	4.24	4.31	4.15	3.83
CE (1:1) / dS m^{-1}		0.08	0.07	0.06	0.04	0.07	0.07	0.06
Organic matter / %		0.84	0.77	3.94	1.68	3.20	2.09	3.13
P / ppm		1.2	2.6	1.4	1.7	2.8	1.9	2.7
K / ppm		96	69	44	68	39	27	45
CEC / meq 100 g		4.80	3.52	17.28	7.68	13.44	9.12	11.52
Exchangeable Cations / meq 100 g	Ca^{2+}	0.33	0.47	0.76	0.42	1.55	1.57	0.45
	Mg^{2+}	0.23	0.23	0.38	0.32	0.50	0.30	0.33
	K^{+}	0.11	0.10	0.17	0.14	0.23	0.15	0.17
	Na^{+}	0.09	0.07	0.09	0.09	0.05	0.09	0.08
	$Al^{3+} + H^{+}$	0.50	0.10	4.90	1.70	2.60	1.30	4.00
Sum of cations		1.26	0.97	6.30	2.66	4.93	3.41	5.03
Sum of bases		0.76	0.87	1.40	0.96	2.33	2.11	1.03
Saturation of bases / %		16	25	8	13	17	23	9
C / %		0.49	1.81	0.45	2.28	1.85	1.21	1.81

TABLE S3 Student *t-test* for comparison of physical-chemical parameters of superficial soil (0–15 cm depth) from clay soil forests (CS) and white-sand soil forests (WS) of the study area

**Soil parameters**	**Total**	**CS**	**WS**	***p*** [a]
	**Mean ± SD**	**Mean ± SD**	**Mean ± SD**	
**Sand percentage**	61.9 ± 19.0	51.4 ± 7.9	88 ± 1.4	<0.001
**Clay percentage**	12.3 ± 4.5	14.4 ± 3.3	7 ± 1.4	<0.001
**pH**	4.2 ± 0.2	4.1 ± 0.2	4.4 ± 0.3	0.245
**Organic matter percentage**	2.2 ± 1.2	2.8 ± 0.9	0.8 ± 0.1	<0.001
**Phosporous ppm**	2.1 ± 0.7	2.1 ± 0.6	1.9 ± 0.9	<0.001
**Potassium ppm**	55.4 ± 23.5	44.6 ± 19.9	82.5 ± 19.1	<0.001
**Carbon percentage**	1.4 ± 0.7	1.5 ± 0.7	1.2 ± 0.9	0.014

[a] t test *p*-value

TABLE S4 Height (cm) of individual plants of *Himatanthus tarapotensis,* per month of evaluation (Apr = April; May = May; Jun = June; Jul = July; Aug = August; Sep = September; Oct = October; Nov = November; Dec = December; Jan = January; Feb = February; Mar = March)

**Plants Specimen Code**	**Months**												**Total**
	**2014**									**2015**			
	**Apr**	**May**	**Jun**	**Jul**	**Aug**	**Sep**	**Oct**	**Nov**	**Dec**	**Jan**	**Feb**	**Mar**	
01	243.6			247.5				251				253	
02	242			277.4				312				360	
03	160.9			163				163				172	
04	276			277.3				302				302	
05	303			306				306				310	
06	150			154.5				183				196	
07	247			260				271				288	
08	175			201				231				238	
09	97			102				124				127	
10	417			460				510				550	
11	288			288				294				297	
12	135			135				135				135	
13	245.5			253				253				257	
14	175			192				208				252	
15	105			111				122				130	
16	122			122				122				122	
17	146.5			146.5				152				156	

TABLE S5 Abundance (number of individuals) of the insect herbivore *Isognathus leachii*, per individual plants of *Himatanthus tarapotensis* and month of evaluation (Apr = April; May = May; Jun = June; Jul = July; Aug = August; Sep = September; Oct = October; Nov = November; Dec = December; Jan = January; Feb = February; Mar = March)

**Plants Specimen Code**	**Months**												**Total**
	**2014**								**2015**				
	**Apr**	**May**	**Jun**	**Jul**	**Aug**	**Sep**	**Oct**	**Nov**	**Dec**	**Jan**	**Feb**	**Mar**	
01	0	9	1	4	4	2	9	1	7	1	0	5	43
02	0	3	0	3	1	2	1	0	13	6	0	0	29
03	0	3	0	1	0	0	2	0	2	6	0	2	16
04	0	5	0	2	2	3	2	1	5	6	0	0	26
05	6	1	0	0	6	26	9	0	0	0	0	0	48
06	0	2	0	0	0	3	10	0	0	0	0	2	17
07	0	0	0	0	5	7	7	8	11	4	0	0	42
08	0	0	0	4	2	1	6	7	2	1	0	0	23
09	0	1	1	0	0	0	1	0	2	1	0	0	6
10	0	2	0	1	0	0	2	0	0	0	0	0	5
11	0	0	0	0	1	3	1	0	0	1	0	0	6
12	0	1	0	1	7	2	0	1	0	1	0	0	13
13	0	1	1	3	15	1	0	0	1	1	0	0	23
14	0	0	0	0	0	0	0	0	0	0	0	0	0
15	0	0	0	0	0	0	0	0	3	1	0	0	4
16	0	0	0	1	0	0	0	0	0	0	0	0	1
17	0	0	0	0	0	0	0	0	1	6	0	0	7
